# Multivariate Regression Analysis to Predict Postoperative Refractive Astigmatism in Cataract Surgery

**DOI:** 10.1155/2020/9842803

**Published:** 2020-01-14

**Authors:** Atsushi Kawahara, Tatsuhiko Sato, Ken Hayashi

**Affiliations:** ^1^San Ai Eye Clinic, 1-3-28, Yamauchi, Okinawa 904-0034, Japan; ^2^Hayashi Eye Hospital, 4-23-35, Hakataekimae, Hakata-ku, Fukuoka 812-0011, Japan

## Abstract

**Purpose:**

To assess the correlation between postoperative refractive astigmatism and preoperative parameters in cataract surgery.

**Methods:**

Left eyes of 100 consecutive patients scheduled for cataract surgery with a 2.4 mm clear corneal incision were examined prospectively. Refractive astigmatism was measured using an autokerato/refractometer. Corneal astigmatism of the total cornea was calculated using a Scheimpflug camera. The vertical/horizontal component (J0) and oblique component (J45) of refractive and total corneal astigmatism were determined using power vector analysis. Refractive astigmatism at 8 weeks postoperatively was estimated using multivariate linear regression analysis. Independent variables analyzed included age, sex, refractive astigmatism, total corneal astigmatism, sphere, intraocular pressure, corneal thickness, anterior chamber depth, lens thickness, axial length, and pupil diameter.

**Results:**

Multivariate regression analysis identified total corneal J0 and age as significant contributors to postoperative refractive J0 (*P* < 0.001 and *P*=0.029, respectively). The standard partial regression coefficients in the multiple regression analysis were 0.59 and −0.16 for total corneal J0 and age, respectively. Significant contributors to postoperative refractive J45 were total corneal J45 and lens thickness (*P* < 0.001 and *P*=0.015, respectively). The standard partial regression coefficients were 0.79 and −0.15 for total corneal J45 and lens thickness, respectively.

**Conclusion:**

These results suggest that preoperative total corneal astigmatism is the most significant predictor of postoperative refractive astigmatism when performing astigmatism correction in cataract surgery.

## 1. Introduction

The aim of modern cataract surgery is not only to restore visual acuity but also to achieve optimal postoperative refraction. As a typical example, the introduction of toric intraocular lens (IOL) technology has made it possible to offer better uncorrected visual acuity to patients with astigmatism. A particular IOL model is selected by assuming that astigmatism is derived entirely from the cornea and lens when the surgeon uses a toric IOL in cataract surgery. If other factors also contribute to astigmatism, they could lead to unexpected postoperative astigmatism. We should, therefore, consider what preoperative parameters are predictive of postoperative refractive astigmatism.

A previous study showed that, in pseudophakic eyes after cataract surgery with an incision of 3.5 mm or more, preoperative keratometric astigmatism was the most significant predictor of postoperative refractive astigmatism in multivariate regression analysis [1]. On the other hand, total corneal astigmatism seems to be more suitable than keratometric astigmatism for the evaluation of postoperative refractive astigmatism as the relationship between refractive and total corneal astigmatism is stronger than that between refractive and keratometric astigmatism in pseudophakic eyes [2]. Currently, advances in technology in cataract surgery have led to a reduction of incision size to 2.4-mm or less [3]. In cataract surgery, smaller incisions induce significantly less corneal astigmatism, corneal shape changes, and corneal surface irregularity than do larger incisions [4]. It is widely accepted that there is a linear statistical relationship between refractive astigmatism and corneal astigmatism [5]. The two main contributory factors in refractive astigmatism are the cornea and lens which is eliminated by cataract surgery. Thus, modern cataract surgery with an incision of 2.4 mm or less is a suitable surgical technique for the evaluation of postoperative refractive astigmatism.

The purpose of our study was to measure the correlation between postoperative refractive astigmatism and preoperative parameters in order to determine the significant predictors of refractive astigmatism after cataract surgery. Descriptions of the aforementioned analysis are few as most existing studies of pseudophakic astigmatism describe the correlation between refractive and corneal astigmatism using univariate regression analysis. To our knowledge, this study is the first to assess the correlation between postoperative refractive astigmatism and preoperative parameters, including total corneal astigmatism, in modern cataract surgery.

## 2. Materials and Methods

This study was a prospective observational cohort study and adhered to the tenets of the Declaration of Helsinki. The study was conducted at the Hayashi Eye Hospital in Fukuoka, Japan. The Institutional Review Board/Ethics Committee of the Hayashi Eye Hospital approved the study protocol, and written informed consent to participate in the study was obtained from all patients after explaining the nature of the study.

### 2.1. Subjects

Consecutive patients who visited the Hayashi Eye Hospital between November 17, 2016, and July 28, 2017, were screened for possible inclusion in the study by a clinical research coordinator. Patient screening was continued until 100 patients were enrolled. The inclusion criteria were left eyes scheduled for phacoemulsification with implantation of an IOL (SN60WF; Alcon Laboratories, Inc., Fort Worth, Texas, USA). The IOL power meeting the focal distance that the patient desired (emmetropia or mild myopia) was calculated using the SRK/T formula. For analysis of data, the overall variance of a sample of measurements combined from both eyes is likely to underestimate the true variance because the variance between eyes is usually less than that between patients. Therefore, only left eyes were included in this study. The exclusion criteria were eyes with any pathology of the cornea, optic nerve or macula; eyes with a lens nucleus harder than grade 4 [6]; eyes with poor mydriasis (<4.5 mm); eyes with a possible zonular dehiscence or pseudoexfoliation; and eyes with a history of surgery or inflammation.

A standard phacoemulsification technique was performed through a 2.4 mm horizontal clear corneal single-plane incision (at the 3 or 9 o'clock meridian). A single-piece acrylic IOL (SN60WF) was implanted in the capsular bag through an unenlarged incision. All incisions were hydrated to aid closure of the incision, and no eye required sutures. The same surgeon performed all cataract surgeries.

### 2.2. Outcome Measures

All eligible patients underwent ocular examinations preoperatively and at 8 weeks postoperatively, and data for corrected distance visual acuity, refractive astigmatism, total corneal astigmatism, sphere, intraocular pressure, corneal thickness, anterior chamber depth, lens thickness, axial length, and pupil diameter was collected. Refractive astigmatism, sphere, and intraocular pressure were measured using an autokerato/refractometer (TONOREFII; Nidek, Gamagori, Japan). Total corneal astigmatism was calculated using a Scheimpflug camera (TMS-5; Tomey, Nagoya, Japan). The TMS-5 includes a rotating Scheimpflug system and a Placido-ring topographer. This device obtains topographic data for the total, anterior, and posterior cornea by merging Placido-ring topography with the Scheimpflug system. The magnitude and meridian of the total corneal astigmatism were determined using simulated keratometric values. The reproducibility of the keratometric values was confirmed in the previous studies [7–9]. Corneal thickness was also measured using the TMS-5. Anterior chamber depth, lens thickness, and axial length were measured using swept-source optical coherence tomography (OCT; IOLMaster 700; Carl Zeiss Meditec AG, Jena, Germany). Pupil diameter was measured using a Colvard pupillometer (Oasis Medical, Glendora, California, USA). Information on age and sex were also extracted preoperatively.

Refractive and corneal astigmatism values were converted to power vector components as described by Thibos et al. [10, 11]. This analysis expresses the vertical (90°)/horizontal (180°) astigmatism component as J0 and the oblique astigmatism component (45° and 135°) as J45. In this representation, as astigmatism is represented in rectangular vector form, conventional scalar methods can be applied to each vector component, which simplifies the mathematical and statistical analyses of astigmatism.

### 2.3. Statistical Analysis

Predictors of the postoperative refractive astigmatic components (J0 and J45) were determined by multivariate linear regression analyses. Preoperative parameters analyzed as independent variables included age, sex, refractive astigmatism (J0 or J45), total corneal astigmatism (J0 or J45), sphere, intraocular pressure, corneal thickness, anterior chamber depth, lens thickness, axial length, and pupil diameter. In the multivariate regression analysis, variables with a partial regression coefficient of *P* value >0.20 were removed using the backward elimination method. Variance inflation factors were calculated to assess multicollinearity. Variables with a variance inflation factor of more than 5 were considered to have excessive collinearity and were excluded. Residual errors, which were differences between the measured and predicted values in the multivariate regression analysis of postoperative refractive J0 and J45, were calculated, and both residual plots were produced. For residual analysis, independence of residual errors was assessed using the Durbin–Watson statistic, and heteroscedasticity was assessed using the White test. A Durbin–Watson statistic in the range of 1.5 to 2.5 indicates independence. Data were analyzed with BellCurve for Excel (version 2; Social Survey Research Information Co., Ltd., Tokyo, Japan). A *P* value <0.05 was considered statistically significant.

## 3. Results

This study enrolled left eyes of 100 patients with a mean age of 69.0 ± 6.3 years. Of these patients, 44 (44%) were male. No patient had any perioperative complication. The mean preoperative and postoperative corrected distance visual acuities were 0.35 ± 0.25 and 0.00 ± 0.07 logarithm of minimal angle of resolution, respectively. The mean vector of preoperative refractive, postoperative refractive, preoperative total corneal, and postoperative total corneal astigmatism was 1.05 diopters (D) axis 90.1°, 0.55 D axis 89.8°, 0.43 D axis 90.0°, and 0.17 D axis 89.5°, respectively. Out of a total of 100 patients, 60 (60%) had preoperative refractive astigmatism with 1.0 D or more of magnitude, which is known to significantly deteriorate uncorrected visual acuity in pseudophakic eyes [12]; 26 (26%) had postoperative refractive astigmatism; 20 (20%) had preoperative total corneal astigmatism; and 17 (17%) had postoperative total corneal astigmatism.

Table 1 shows the measurements of variables for multivariate regression analysis. Preoperative refractive, postoperative refractive, and preoperative total corneal astigmatism reflected a small amount of against-the-rule astigmatism on average (J0 < 0). In addition, there was less oblique astigmatism, as indicated by the absolute values of the smaller mean and standard deviation for J45.

Table 2 shows the effects of preoperative parameters on postoperative refractive J0, based on multivariate regression analysis. Age, refractive J0, total corneal J0, intraocular pressure, and corneal thickness were selected as independent predictors of postoperative refractive J0 (adjusted *R*^2^ = 0.55, *P* < 0.001), and the multivariate model did not reveal any problems regarding multicollinearity. Age and total corneal J0 were statistically significant (*P*=0.029 and *P* < 0.001, respectively).

Table 3 shows the effects of preoperative parameters on postoperative refractive J45, based on multivariate regression analysis. Total corneal J45 and lens thickness were selected as independent predictors of postoperative refractive J45 (adjusted *R*^2^ = 0.63, *P* < 0.001), and the multivariate model did not reveal any problems regarding multicollinearity. Both parameters were statistically significant (*P* < 0.001 and *P*=0.015 for total corneal J45 and lens thickness, respectively).

**Figure 1:**
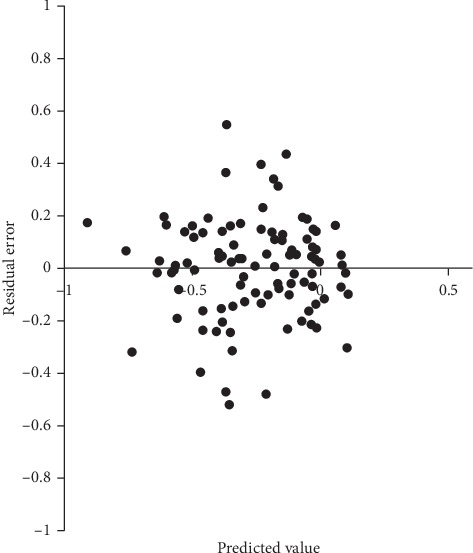
Residual plots of postoperative refractive J0 in multivariate regression analysis.

**Figure 2:**
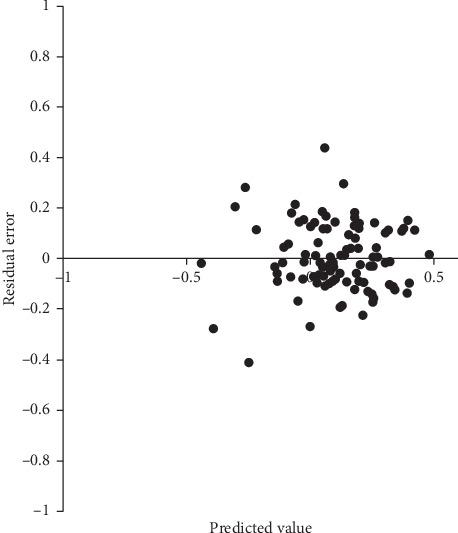
Residual plots of postoperative refractive J45 in multivariate regression analysis.

Residual plots are shown in Figure 1 for postoperative refractive J0 and in Figure 2 for postoperative refractive J45. Both residual plots revealed a random distribution pattern. Independence was observed among the residual errors of postoperative refractive J0 and J45 (Durbin–Watson statistic = 1.728 and 2.060, respectively), but heteroscedasticity was not (White test, *P*=0.641 and *P*=0.071, respectively). These results indicated that the multivariate regression models in this study were valid.

## 4. Discussion

Using multivariate regression analysis, we have examined the relation between postoperative refractive astigmatism and preoperative parameters in cataract surgery with an incision of 2.4 mm. Multivariate regression analysis revealed that preoperative total corneal astigmatism and younger age were significantly correlated with postoperative refractive astigmatism in the vertical/horizontal component and that preoperative total corneal astigmatism and thinner lens were significantly correlated with postoperative refractive astigmatism in the oblique component. The standard partial regression coefficients of preoperative total corneal astigmatism gave the maximum absolute values in the respective analyses. These results suggest that preoperative total corneal astigmatism is the most significant predictor of postoperative refractive astigmatism. Although the absolute value of the standard partial regression coefficient was small, the association of postoperative vertical/horizontal astigmatism with age reflected the well-known change from with-the-rule astigmatism to against-the-rule astigmatism with advancing age [13–16]. Meanwhile, the reason for the association of lens thickness with postoperative oblique astigmatism is unclear. Refractive astigmatism after cataract surgery can be estimated based on the results of this study.

The previous studies demonstrated that refractive and total corneal astigmatism in pseudophakic eyes after cataract surgery with a 2.8 mm or more incision had a weak correlation in univariate regression analysis (*R*^2^ values: 0.13 to 0.36) [2, 17]. A more recent study showed a strong correlation in pseudophakic eyes after cataract surgery with a 2.0 mm incision (*R*^2^ values: 0.70 to 0.85) [18]. Pseudophakic refractive astigmatism is more likely to be better correlated with corneal astigmatism in cataract surgery using smaller incisions. Leffler et al. reported that refractive astigmatism after cataract surgery with an incision of 3.5 mm or more and preoperative keratometric astigmatism were correlated (*R*^2^ values: 0.51 and 0.05 for J0 and J45, respectively) and preoperative keratometric astigmatism was the most significant predictor in multivariate regression analysis using postoperative refractive astigmatism as a dependent variable and preoperative parameters as independent variables. In contrast, the present study demonstrated that postoperative refractive and preoperative total corneal astigmatism had a better correlation (adjusted *R*^2^ values: 0.55 and 0.63 for J0 and J45, respectively) and preoperative total corneal astigmatism was the most significant predictor. This was probably due to the smaller incision width (2.4 mm incision) and the evaluation of the total cornea in our study. Newer technologies are now available for measuring total corneal astigmatism based on both the anterior and posterior corneal shapes [19]. Keratometric astigmatism is taken from points in the central anterior cornea and estimates the total corneal power based on a reduced refractive index; that is, an averaged corneal refractive index that takes into account the anterior surface, the different layers of cornea, and the negative power of corneal back surface, which are not actually measured. Keratometric astigmatism differs significantly from total corneal astigmatism based on both anterior and posterior corneal measurements [19, 20]. This is based on the assumption that the refractive power of the posterior cornea is proportional to that of the anterior cornea. Recent studies, however, revealed that the posterior corneal astigmatism is not necessarily proportional to the anterior corneal astigmatism [19, 21–23]. Accordingly, it is necessary to measure the total corneal astigmatism to evaluate corneal astigmatism.

The current study had a number of limitations. First, a Placido–Scheimpflug system was used for the evaluation of the anterior and posterior cornea and we did not use swept-source anterior-segment OCT. As swept-source OCT measures the corneal curvature of the anterior and posterior cornea based on one principle, measurements are thought to be more accurate than those of the Placido–Scheimpflug system [24]. Second, 80% of our enrolled patients had a corneal astigmatism magnitude of 1.0 D or less although patients for toric IOL implantation generally have considerable corneal astigmatism. Third, only left eyes were analyzed in this study. However, the findings of this study should be applicable to right eyes as refractive astigmatism [25–28], corneal curvature [25, 29–32], and pupil center [33] demonstrate mirror interocular symmetry about the midsagittal plane. Fourth, the present study is the relatively short follow-up duration. Corneal shape changes rapidly diminish and stabilize within 2 months postoperatively, however, when the incision width is 2.4 mm or less [34]. When the incision width is 2.4 mm or less, a longer follow-up may not be necessary. Additional research should be conducted to clarify these points.

## 5. Conclusions

Our findings indicate that preoperative total corneal astigmatism is the most significant predictor of refractive astigmatism after modern cataract surgery. It is appropriate to use the measurement of total corneal astigmatism for evaluation prior to correcting astigmatism during cataract surgery. However, our findings warrant further investigation to find causes of residual errors beyond the preoperative parameters of this study.

## Figures and Tables

**Table 1:** Variables for multivariate regression analysis.

	Dependent variables		Independent variables (preoperative parameters)												
	Postoperative refractive J0 (D)	Postoperative refractive J45 (D)	Age (years)	Sex (men/women)	Refractive J0 (D)	Refractive J45 (D)	Total corneal J0 (D)	Total corneal J45 (D)	Sphere (D)	Intraocular pressure (mmHg)	Corneal thickness (mm)	Anterior chamber depth (mm)	Lens thickness (mm)	Axial length (mm)	Pupil diameter (mm)
Mean ± SD	−0.25 ± 0.29	0.11 ± 0.22	69.0 ± 6.3	44/56	−0.52 ± 0.45	−0.09 ± 0.33	−0.21 ± 0.26	0.02 ± 0.21	−1.31 ± 4.12	14.1 ± 2.8	0.537 ± 0.031	3.30 ± 0.36	4.69 ± 0.66	24.33 ± 1.46	3.59 ± 0.52
Range	−1.06–0.30	−0.67–0.54	47–80		−2.61–0.25	−1.31–0.89	−1.07–0.37	−0.65–0.44	−21.50–3.50	9.0–22.0	0.455–0.655	2.28–4.16	3.44–7.51	21.82–30.67	3.0−5.0

**Table 2:** Multivariate regression analysis of postoperative refractive J0.

Preoperative parameters	Partial regression coefficient	*P* value	Standard partial regression coefficient
Age	−0.01	0.029	−0.16
Refractive J0	0.10	0.054	0.16
Total corneal J0	0.65	<0.001	0.59
Intraocular pressure	0.01	0.055	0.14
Corneal thickness	−1.27	0.066	−0.14
Intercept	0.90	0.020	

Adjusted *R*^2^ = 0.55, *P* < 0.001.

**Table 3:** Multivariate regression analysis of postoperative refractive J45.

Preoperative parameters	Partial regression coefficient	*P* value	Standard partial regression coefficient
Total corneal J45	0.82	<0.001	0.79
Lens thickness	−0.05	0.015	−0.15
Intercept	0.33	0.001	

Adjusted *R*^2^ = 0.63, *P* < 0.001.

## Data Availability

The data used to support the findings of this study are restricted by the Hayashi Eye Hospital Ethics Committee in order to protect patient privacy and can be obtained upon request to the corresponding author (atsusi-k@coral.plala.or.jp).
